# Perceptions, health seeking behavior and utilization of maternal and newborn health services among an indigenous tribal community in Northeast India—a community-based mixed methods study

**DOI:** 10.3389/fpubh.2023.1139334

**Published:** 2023-07-06

**Authors:** Ángela León Cáceres, Rohan Michael Ramesh, Puisaumaliu Newmai, Rhondemo Kikon, Andreas Deckert

**Affiliations:** ^1^Heidelberg Institute of Global Health, University of Heidelberg, Heidelberg, Germany; ^2^Tropical Herping, Quito, Ecuador; ^3^Christian Institute of Health Sciences and Research, Dimapur, India; ^4^Community Health Initiative, Dimapur, India

**Keywords:** maternal and newborn health, health seeking behavior, tribal, access to health services, health service utilization, social determinants of health, universal health coverage (UHC), India

## Abstract

**Background:**

Evidence suggests that healthcare utilization among tribal communities in isolated regions can be influenced by social determinants of health, particularly cultural and geographical factors. The true mortality and morbidity due to these factors in remote tribal communities are often underestimated due to facility-dependent reporting systems often difficult to access. We studied the utilization of health services for maternal and newborn care and explored how cultural beliefs, perceptions, and practices influence the health-seeking behavior (HSB) of an indigenous tribal community in Northeast India.

**Methods:**

Within a concurrent triangulation design, the combined results from 7 focus group discussions and 19 in-depth interviews, and the 109 interviews of mothers from a community-based survey were interpreted in a complementary manner. The qualitative data were analyzed using a conceptual framework adapted from the socio-ecological and three-delays model, using *a priori* thematic coding. Multivariable logistic regression was carried out to identify factors associated with home delivery.

**Results:**

Only 3.7% of the interviewed mothers received the four recommended antenatal check-ups in health centers, and 40.1% delivered at home. Mothers residing in the villages without a health center or one that was not operational were more likely to deliver at home. HSB was influenced significantly by available finances, the mother’s education, low self-esteem, and a strong belief in traditional medicine favored by its availability and religious affiliation. The community sought health services in facilities only in emergency situations, determined primarily by the tribe’s poor perception of the quality of health services provided in the irregularly open centers, locally available traditional medicine practitioners, and challenges in geographical access. National schemes intended to incentivize access to facilities failed to impact this community due to flawed program implementation that did not consider this region’s cultural, social, and geographical differences.

**Conclusion:**

The health-seeking behavior of the tribe is a complex, interrelated, and interdependent process framed in a medical pluralistic context. The utilization of health centers and HSBs of indigenous communities may improve when policymakers adopt a “bottom-up approach,” addressing structural barriers, tailoring programs to be culturally appropriate, and guaranteeing that the perceived needs of indigenous communities are met before national objectives.

## Introduction

The global health agenda has emphasized decreasing maternal and newborn mortality as key components of sustainable human development. Despite the progress, 830 women die every day from causes related to pregnancy or childbirth; of this, 20 percent are from India (1, 2). This country still has significant challenges, even if the maternal mortality ratio was reduced from 212 deaths per 100,000 live births in 2007 to 167 deaths in 2013 (3). However, in 2013, the highest number of neonatal deaths globally (0.75 million) was reported in India (4). Further, within the country, the neonatal mortality rate (NMR) nearly doubles in some rural and tribal areas compared to the urban areas since they face considerable inequalities in access to antenatal, intranatal, and postnatal healthcare services (4). Other reasons that contribute to this disparity in India are low public expenditure on health (1.02% of India’s Gross Domestic Product for 2015–2016), high dependency on private healthcare services (78%-outpatients and 60%-inpatients), and high out-of-pocket (OOP) expenditures especially in Nagaland (61.7%) compared to the rest of India (20.5%) (5–7). To address the aforementioned issues, the Indian Government launched the “Janani Shishu Suraksha Karyakaram” (JSSK) scheme in 2011 for pregnant women and newborns to decrease OOP and neonatal deaths by providing free transport, medicines, and consumables, diagnostic tests, blood transfusions, and food for the duration of a woman’s stay. Sick neonates are also entitled to free treatment and transport (5). Additionally, another program, the “Janani Suraksha Yojana” (JSY) program offers cash assistance to mothers and accredited social health activists (ASHA-local community health volunteers who promote access to health services, mobilize communities, and provide community care) to facilitate institutional deliveries (8).

The Northeast region of India accommodates the country’s largest proportion of scheduled tribes (ST) and ranks the lowest in social indicators (9). One such state in this region is Nagaland, where 61% of the people are multidimensionally poor and live in rural and remote zones that lack connectivity due to inadequate or non-existent public transport systems, which markedly influences timely, affordable, and accessible maternal and child public healthcare services (10). In 2016, the more developed states like Kerala and Tamil Nadu reported almost 100% institutional deliveries, but Nagaland reported only a third of its births in health facilities (11). Nearly 90% of the population living in Nagaland are tribal or indigenous. “Nagas” is a collective term for several indigenous communities in the Northeast Region of India and upper Myanmar that share similar social and cultural characteristics. In India, the majority of the Nagas live in the state of Nagaland, within 16 administrative districts inhabited by 17 major and sub-tribes. In India, Nagas are classified as a scheduled tribe (Indigenous people from India who are officially regarded as socially disadvantaged) under the Indian constitution article 342 (12).

While there is anecdotal evidence suggesting that political unrest, the rugged terrain, and the lack of transport greatly influence access to healthcare services, a paucity of data demonstrates that social and cultural factors also influence health-seeking behavior (HSB) exhibited by this community. This study employed a mixed methods approach to quantitatively study the utilization of maternal and newborn health services while adopting a salutogenic perspective to explore how cultural beliefs, perceptions, and practices influence the HSB of an indigenous tribal community in Northeast India.

## Materials and methods

### Study setting

**Figure 1:**
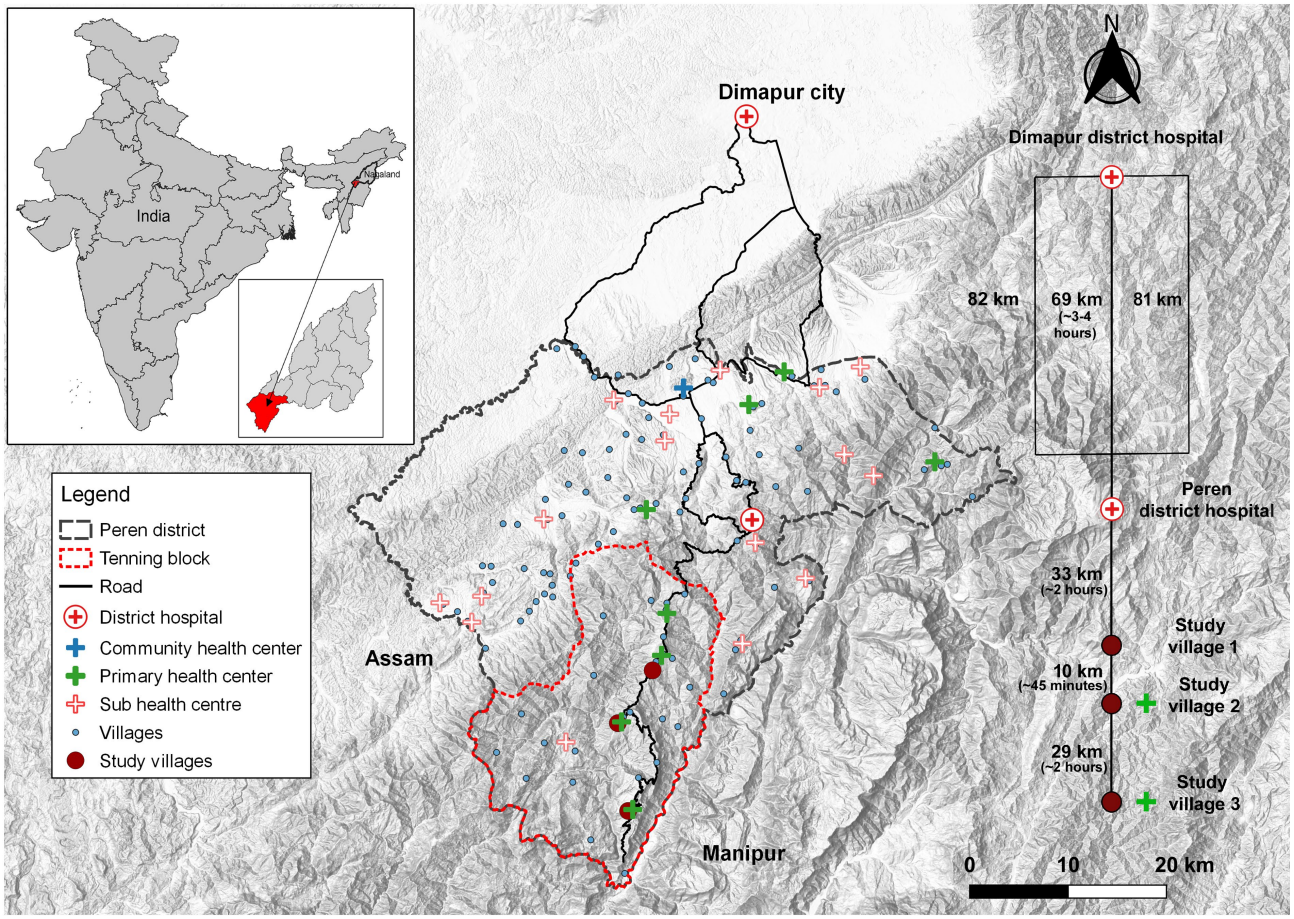
A topographic map of the study area with details of access to health facilities.

The study was conducted in Tening, a block in Peren district, located in the hilly north-eastern state of Nagaland, India. The community’s total population is around 95,000, and the Tening block consists of 23 registered villages, currently home to the Christianized Liangmai Naga tribe (also called “Zeliang”, one of the 17 major tribes in Nagaland) (13). Due to civil conflicts, this territory became a no-go area in 1947, was a restricted area for more than 50 years, and is presently considered a disturbed area, the consequences of which still disrupt everyday life (14). Their lifestyles and social structures are based on a lineage and patriarchal clan system. The Northeastern region of India presents a complicated geographical picture that isolates this region from the rest of the country. The Tening block is landlocked by the neighboring states of Manipur in the southeast and Assam in the southwest. Dimapur is the closest city with tertiary healthcare facilities located ~120 km from the block in the adjacent district; however, it takes nearly 6–8 h to reach there due to the poor condition of the roads, especially during the rainy season (Figure 1). Accessibility is further complicated due to the high costs expected by local private transport agencies in the absence of a functional public transport system. Around 80% of the population is engaged primarily in agricultural activities (15). In 2016, the district reported that 41% of the mothers delivered in health centers, and only 13% of pregnant women received the recommended ≥4 antenatal care (ANC) checkups in a facility (16). In 2018, the government reported four stillbirths, nine infant deaths, and zero maternal deaths during the same period (17). Of the four primary health centers (PHC) and one Health sub-centre (HSC) located in the Tening block, only one PHC is fully functional as a delivery point. The structure of the Indian public health system has been described in detail elsewhere (18). Obstetric and neonatal emergencies are referred to the District hospital located in the adjacent block, but due to the lack of facilities to manage complications, they are further referred to tertiary centers located in Dimapur city (Figure 1).

### Study design

A mixed-methods approach with a concurrent triangulation design was used to capture qualitative and quantitative maternal and newborn health information from the participants residing in the study villages. Three villages from the block were selected after a pilot visit based on the presence and functionality of a PHC in the village. Village 1 did not have a health facility inside the village and the closest functional delivery point is the PHC is located in Village 2. Village 2, has a fully operational PHC in the village and also caters to the entire block. Village 3 was the furthermost village; although there was a physical structure the PHC was not-operational (Figure 1).

### Conceptual framework

We explored the HSB of the tribe from a holistic and community-centered perspective based on a theoretical framework that assumes that perceptions are subjective and thus guided by social actions, which can be constructed or deconstructed individually and socially, with an emphasis on the effect of social interactions, relations, and language conventions. This study considered pregnancy and childbirth as social and cultural events guided by different nested norms in a pluralistic medical system. The socio-ecological model (SEM) explored cultural beliefs and practices influencing the decision-making process and perceptions of the provided services. The SEM offers a multi-level approach to understanding the interaction and interdependence of the individual, interpersonal, organizational, community, and public policy levels (19). Furthermore, it includes the individual’s perceptions, cultural beliefs, and knowledge about the issue under investigation. At the organizational level, the model seeks to interpret the reciprocal causation between people’s considerations about the access and utilization of these services in the given environmental circumstances to explore possible barriers that are perceived to influence community and individual behaviors (19). To describe the barriers that might affect access and utilization of maternal and newborn healthcare services and the process of seeking them, we constructed a conceptual framework based on the *three delays model* (20). We adapted the model, which originally addressed the factors that affect obstetric complications and explored the *delays* in access to ANC, perinatal care services, postnatal care (PNC), and neonatal care while considering relevant factors that influence maternal and newborn mortality and morbidity. The first and second delays: (1) delay in the decision to seek care, and (2) delay in arrival to the facility, were especially emphasized (20). For the perceptions of the tribe regarding the government’s services, the third delay offered a suitable framework: (3) delay in the provision of adequate care (20).

### Data collection

*Qualitative* data was collected through focus group discussions (FGDs) and in-depth interviews (IDIs) conducted between March 23 – April 19, 2019. To *triangulate sources* and consider the cultural context in which the study was developed, we selected participants all above 18 years of age from five different groups of the Liangmai tribe: (1) women of childbearing age, (2) male relatives/partners of women of childbearing age, (3) traditional birth attendants (TBA), (4) or ASHAs/ community health workers (CHWs) who are currently residing in each village, and (5) health managers or health professionals at the government health facilities. The cross-lingual IDIs and FGDs were conducted in groups of 5–6 participants and were facilitated by a bilingual moderator.

The *quantitative* part comprised information from an extensive district-level community-based cross-sectional survey studying the influence of geographical variation on maternal and child health events conducted between November 2018 and December 2019 from 70 villages in the Peren district. In brief, all mothers who delivered in the last 2 years, regardless of the outcome and residing in the study villages, were approached to participate in the in-person interviews. After obtaining the informed consent, the trained field interviewers administered a questionnaire to collect the antenatal, intranatal, and postnatal details from the mother. Details of the child were also collected while corroborating the information provided using the “mother and child tracking card” provided by the government to all mothers, when available. Waypoints of the mother’s house and routes to the nearest health facility were mapped. For this study, the data from the 3 study villages using a subset of relevant questions were extracted to analyze and corroborate with the findings from the *qualitative* component. The aim of the quantitative part was to describe the scenario rather than testing a certain scientific hypothesis; hence, we waived a sample size calculation and instead used data from the full study population (all mothers who delivered in the last 2 years) to get more precise estimates. Data from the community-based survey: Thirteen pertinent questions from the cross-sectional survey (demographic details, obstetric history, antenatal care, pregnancy outcome, healthcare workers visits, details of the delivery, and transportation) were selected to corroborate with the qualitative demographic questionnaire, the FGD, and the IDI guides.

### Data collection tools

The qualitative data collection tools included semi-structured cross-lingual FGDs and IDIs, adapted to the different target groups and supported by participant observation. In the FGDs, *free listing* and *pile sorting* techniques were applied to identify which health conditions people were seeking healthcare services for and to prioritize the intensity or severity of their thoughts. This was used to systematically identify robust group schemes and shared knowledge, beliefs, and perceptions. IDIs explored the individuals’ perceptions, experiences, knowledge, beliefs, practices, and behaviors. The questions were also meant to guide the participants to reflect and perceive themselves as community members and to understand the individual and their families within the structure of the macro system. In this cultural setting, we arranged the groups by sex and similar ages to understand their perspectives, obtain information about the social norms, group behaviors, hierarchies, power relations, degree of consensus, and demonstrate group emotional processes.

Additionally, we wanted to understand the group’s influence on an individual and how or in what ways it influences individuals. To facilitate communication with the community, a trained research assistant operated as a bilingual moderator and interviewer in the FGDs/IDIs, and also as an interpreter and translator in the informal conversations with key informants. The principal researcher trained the research assistant before the study on qualitative methods, tools, ethical considerations for research, social determinants of health, and basic concepts of maternal and newborn health. A problem tree analysis was performed to contextualize the problem and study hypothesis. A document with key terms was formulated during the training to guide the interviewer in obtaining answers from the participants.

Semi-structured interview guides were developed for the interviews to avoid potential personal bias. The principal researcher was a passive observer, taking notes of the body language of the participants and occasionally assisting the interviewer. IDIs were used to explore the perceptions, experiences, knowledge, beliefs, practices, and behaviors of the participants. The IDI guides were selected as an appropriate tool to ensure consistency during the interviews and facilitate the analysis process, tailored for each sub-group. FGD as a qualitative tool was chosen understand the perceptions of the group in its social setting and collecting several perspectives on the topic. FGDs helped to obtain information about the social norms, group behaviors, hierarchies, power relations, degree of consensus, and demonstration of group emotional processes. The FGD and IDI guides, the information-sheet, and informed consent forms (for the FGD, IDI, demographic questionnaire, and photograph use) were developed in English and were translated into Nagamese and Liangmai. The FGDs and IDIs were primarily conducted in the local language (Liangmai and Nagamese). No FGD participants were interviewed in the IDIs.

The quantitative data from the survey were collected by a trained interviewer using a mobile-based, semi-structured, translated, pilot-tested questionnaire. The pilot questionnaire was administered in the local language to 12 tribal mothers who had delivered in the last 2 years residing in a non-study village in the adjacent district. When the study commenced, the field interviewer approached a mother and invited her to participate after providing an information sheet printed in the local language. If the mother could not read, the field interviewer was instructed to read it out in the local language. Any queries regarding the study were discussed before obtaining the informed consent sheet if she decided to participate.

### Data analysis

Four of the 26 audio-recorded sessions were transcribed verbatim into Liangmai and then translated to English, while the rest were translated directly into English. Additional revisions of the translations were done in collaboration with an external Liangmai native speaker, assuring confidentiality. The study used a combination of both deductive and inductive approaches to coding. The English transcripts were coded *a priori* according to specific content priority analysis. Two coding approaches were used: *concept-driven* and *data-driven coding*; then, the data were analyzed using NVivo 12 Pro [QSR International Pty Ltd., (2018) NVivo (Version 12)]. The codes used were related to the conceptual framework, guiding principles of the study, and social determinants of health. The codes were related to the adapted Three Delays Model in order to describe the barriers that might affect the access and utilization of maternal and newborn healthcare services and the process of seeking them. A special emphasis was put on the first and second delays: (1) delay in the decision to seek care; (2) delay in arrival to the facility. For perceptions of the tribe regarding the government’s services, the third delay; and (3) delay in providing adequate care offered the suitable framework. This was combined with the SEM, because it offered a multiple-level approach to understanding the interaction and interdependence of the individual, interpersonal, organizational, community, and public policy levels.

Emphasis was placed on cultural determinants, barriers to accessing or utilizing healthcare services, motivational determinants, decision-making processes, and linkages between Traditional Medicine (TM) and government healthcare services, human, and interpersonal relationships A qualitative multilevel data analysis approach was applied, complementing it with an analysis of the observation notes and the free-listing and pile sorting techniques conducted in FGDs. We described the characteristics of the FGDs and IDIs participants, using frequencies for age, marital status, education, and occupation, stratified by gender.

Quantitative data: The frequencies for categories of all survey variables were calculated. Additionally, simple descriptive statistics with fisher’s exact test were used to investigate if there were significant differences in each demographic variable between the study villages. The significance level alpha was set at 5%.

To adjust for potential confounders, variables from the univariable analysis were chosen for a multivariable logistic regression model in (i) a full model and (ii) a model with backward selection to study the factors associated with home delivery, using SPSS 21 (IBM Corp. Released 2012. IBM SPSS Statistics for Windows, Version 21.0. Armonk, NY: IBM Corp). The outcome variable was the place of delivery. The backward selection started with all variables included except for marital status (only one unmarried person), the person responsible for the delivery (directly related to the outcome variable), and fully immunized children (immunization was beyond the time point of delivery). The final model consisted of only the villages.

### Ethics approval statement

The ethical approval was obtained from the Ethics Committee of the Medical Faculty, the Ruprecht Karls University of Heidelberg, Germany (#S-138/2019, 13.03.2019), and from the Institutional Review Board (Research and Ethics Committee) of the Christian Institute of Health Sciences and Research, Nagaland, India for the qualitative component and the quantitative data from the cross-sectional survey from the “Project on the use of Geographic Information Systems in Health Systems Research in Rural Areas of Nagaland”. Approved data collection tools were translated into Nagamese and Liangmai languages and submitted to the Institutional Review Board of the Christian Institute of Health Sciences and Research. Written informed consent was obtained from all participants.

## Results

A total of 57 community members participated in the seven FGDs (four FGDs with women of childbearing age and three FGDs with male relatives/partners) and 19 IDIs (five with women of childbearing age, four with male partners or relatives of women of childbearing age, four with the ASHAs, four with TBAs and traditional healers, and two with health professionals). Responses from interviews with 109 mothers residing in the 3 study villages were included in the cross-sectional survey.

### Characteristics of the survey participants

Of the 109 women who participated in the cross-sectional survey, most (73.4%) were 20 to 29 years of age, with a mean age of 27 years, and married. The majority (46.8%) of the mothers had completed up to middle school education, while 21.1% had completed only up to primary education, and 5.5% were graduates or held higher educational degrees. Most (45.9%) mothers were housewives and farmers (41.3%), but this differed between the three villages (*p* < 0.01) as farming was the preferred vocation in villages 1 and 3 (Supplementary Table S1).

### Characteristics of the FGD and IDI participants

The demographic information collected from the female participants in the FGDs and IDIs portrayed a similar picture. Most 80.0% (*n* = 28) of the women were currently married, 63.6% of them had completed only up to primary education, 6% held a university degree, and the majority of them were housewives (20%) and farmers (34%). Most (76.2%, *n* = 16) participating males were married, 20.0% had university or higher degrees, and 52.9% worked as farmers (Supplementary Table S2).

### Obstetric details

**Figure 2:**
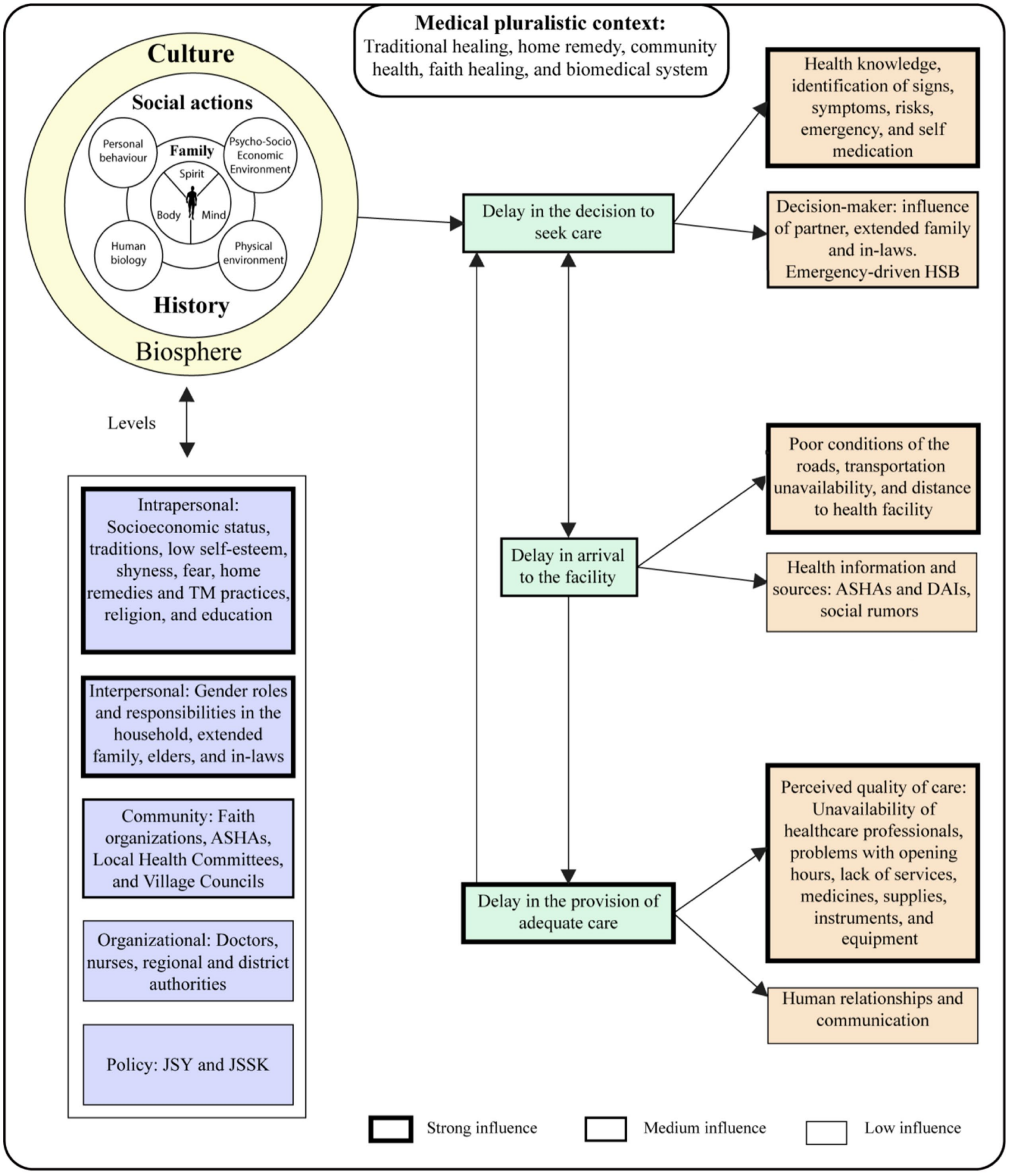
Main determinants and their level of influence regarding the SEM and the adapted-three delays model.

The cross-sectional survey revealed that 56.0% (61/109) of the mothers had experienced 2–4 pregnancies, while 22.0% (24/109) had up to five or more pregnancies. Only 3.7% (4/109) had completed atleast the four recommended ANC check-ups, and 11.9% (13/109) of the mothers did not receive even one ANC check-up. Around 14.7% (13/109) of the mothers received just one, while most (69.7%, 76/109) received at least 2–3 check-ups in a health facility. The proportion of mothers receiving nil or just one ANC check-up was higher in the village with no health center (village 1) compared to the other two. The difference in proportions between the three villages was statistically significant (*p* < 0.01). Some (11%, 9/109) mothers reported at least one spontaneous abortion, and 14.7% (16/109) reported 1–2 newborn or infant deaths in the near past. The majority (40.4%, 44/109) of mothers delivered at home, while 41.3% (45/109) at the local PHC located inside the block, 13.8% (15/109) at a public health facility located outside the block, and 4.6% (5/109) in private facilities outside the block. Most (50%) mothers residing in the village with a functional delivery point (village 2) preferred to deliver in a facility hence the proportion of home deliveries was the lowest (22.6%) of the three. Though village 1 did not have a health facility located in the village, many mothers (41.3%) chose to deliver at the closest local PHC located in village 2 (Figure 1). However, 73.9% of the mothers residing in the furthermost village with a non-operational health center (village 3) delivered at home, while only 17.4% of the mothers delivered in a facility that was located 2–3 h away because the delivery point the village was mostly nonfunctional (Figure 2). A statistically significant difference was noticed in the place of delivery between the three villages (*p* < 0.01). The majority of the deliveries were attended to by a nurse (64.2%), and the rest were attended to by relatives (22.9%), ASHAs (10.1%), TBAs (0.9%), and only 1.8% by a doctor. Most (83.9%) women residing in the village with a functional delivery point informed that nurses conducted the deliveries. In comparison, only 41.7% of women from the village with no health center (Village 1) reported the same. The difference in the person responsible for the deliveries between the three villages was statistically significant (*p* < 0.01; Supplementary Table S1). Most women (61.3%, 19/31) who chose to deliver in the functional health facility inside their village (Village 2) walked to the delivery point. At the same time, the majority (54.5%, 12/22) who decided to deliver in health facilities outside the block hired a local taxi, and one respondent reported using an ambulance. The multivariable logistic regression model using backward selection revealed that mothers residing in Village 1-without a health center [adjusted odds ratio (aOR), 4.1, 95% CI, 1.5–11.0] and especially Village 3-with a non-operational health center (aOR,9.7, 95%CI, 3.2–29.3) were more likely to deliver at home (Supplementary Table S3).

### Beliefs, practices, behaviors, and perceptions influencing health-seeking behavior

The SEM was adapted to understand the factors influencing decision-making that affect individual HSBs and their perceptions regarding healthcare facilities. They are subdivided according to the order of influence into three levels.

#### The intrapersonal and interpersonal level

This level had the most decisive influence on an individual’s perceptions and HSB, as illustrated in Figure 2. The qualitative component identified socio-economic status, education, low self-esteem, shyness, fear, traditions, home remedy/TM practices, and religion immensely influence the individual’s HSB. Most participants stated they could not access healthcare services, including preventive health and medicines, due to the lack of money and their own-self denomination as “poor” (Table 1). The gender disparity in educational levels may also influence decision-making (40% of men with university education compared to 6% of women). The interviewed women showed low confidence and self-esteem; most indicated anxieties, suffering, pain, and fear of pregnancy, especially childbirth (Table 1). These were guided by the idea of dying or watching a partner die during delivery. According to the TBAs, most women in this community expressed ‘shyness’ when deciding on or visiting health facilities. Health professionals also corroborated that most women were shy and reluctant to be treated by a male doctor, compelling them to seek care from female nurses.

**Table 1:** Highlights from the focus group discussions and in-depth interviews included in the socio-ecological model.

Level on the socio-ecological model	Factors	Quotes
The intrapersonal and interpersonal level	Socioeconomic status	*“While pregnant and to take her, the biggest obstacle would be money. If there is no money how do we take? Many want to go but if there is no money, how can we? That’s the biggest difficulty we face in village. Those who have, they can, but those who do not then that’s the problem and that’s how they do not take them and deliver at home.”- Liangmai man, 38 years old*
	Sense of confidence and self-esteem	*“Me, I am not clever, and we are nothing right?” – Liangmai woman, 35 years old*
	Fear of the individual and the community	*“So, for women, giving birth is the toughest thing. It is like sitting on the edge of the cemetery.” – Liangmai woman, 27 years old*
	Traditions and myths	*“My grandmother always said: you should not go anywhere, and you do this and that ok? (…) When we are pregnant our stomach should be properly hidden. Our stomach should not be open. That is the advice of the elders which we know, from our experience. Means, they say that bad things are seen through it. When we get pregnant our stomach should be covered very nicely and should not be allowed to be seen.” – Young Liangmai pregnant woman*
		*“Giving birth is a battle of life, during delivering whether a woman will live or die. Before our forefathers left us with this saying. A pregnant or women after deliveries should be taken good care because they fight for their lives and whether they live or die they do not know.” – Liangmai male partner, 38 years old*
	Religion and trust kinships	*“Because when we go to doctors and they cannot do, we ask God for the help and His direction. Because God knows everything. So, God shows the direction on how to go and do.” – Liangmai woman, 28 years old*
		*“More than doctors. I trust God more. Prayers.” – Liangmai woman, 31 years old*
	Family and spouse	*“If a woman is getting a baby, then the full responsibility should be on the father, a husband should take care of everything.” – Liangmai husband, 30 years old*
	Perception of biomedical services	*“So we will go to the hospital… that’s the last resource or else no other way.” – Liangmai man, 24 years old*
	Role of family as sources of knowledge	*“We get advice from the elder people. Like from my mom and dad. What’s the best thing(s) that we should do? Like for our health issues. They will just give us the advice: “Do like that.” And then we will go” – Liangmai man, 30 years old*
The community, organizational, and policy level	Community health workers	*“When pregnant, our families, husbands also, or brothers-sister… suppose, to me, I should not go alone. ASHA is there, colony’s ASHA is there. ASHA takes you and will take you (referring to the health facility)” – Liangmai woman, 35 years old*
	Faith based organizations	*“We, after birth do not have anything to do…We take it to the church, we do this only” – Liangmai woman, 37 years old*
	Public health strategies	*“In our report, we write for more hospital deliveries. Home delivery is more, but because the women wants to get the money, because we they are told to give birth in the PHC. It is difficult for us to take them also. So, even if the delivery is at home we write as delivery in the hospital. We lie because they will not get money, right? The JSY and all.” – ASHA, 41 years old*
	Health professionals perspective	*“We do not have proper light also, we do not have electricity, we do not have mobile network also, we are facing problems” – Doctor*

Traditions, myths, religion, and perceptions about biomedical services and TM influence the tribe’s HSB. Most participants expressed that their forefathers believed pregnancy and childbirth could endanger the mother’s life (Table 1). They also reluctantly spoke about the myths surrounding the practices of a primordial religious minority community. The latter was associated with black magic and witchcraft, which contradicts their strong faith in Christianity for healing, as evident through the discussions (Table 1). All participants responded that praying and their belief in God will resolve their health issues, indicating that most healing practices, either traditional or biomedical, are accompanied by individual or group praying sessions. Some participants believe that praying protects the baby from “abnormalities”. Strong religious beliefs were also observed in the responses from TBAs, ASHAs, and traditional healers as well. The latter believe their healing is a gift from God and is also achieved through the individual’s faith.

**Figure 3:**
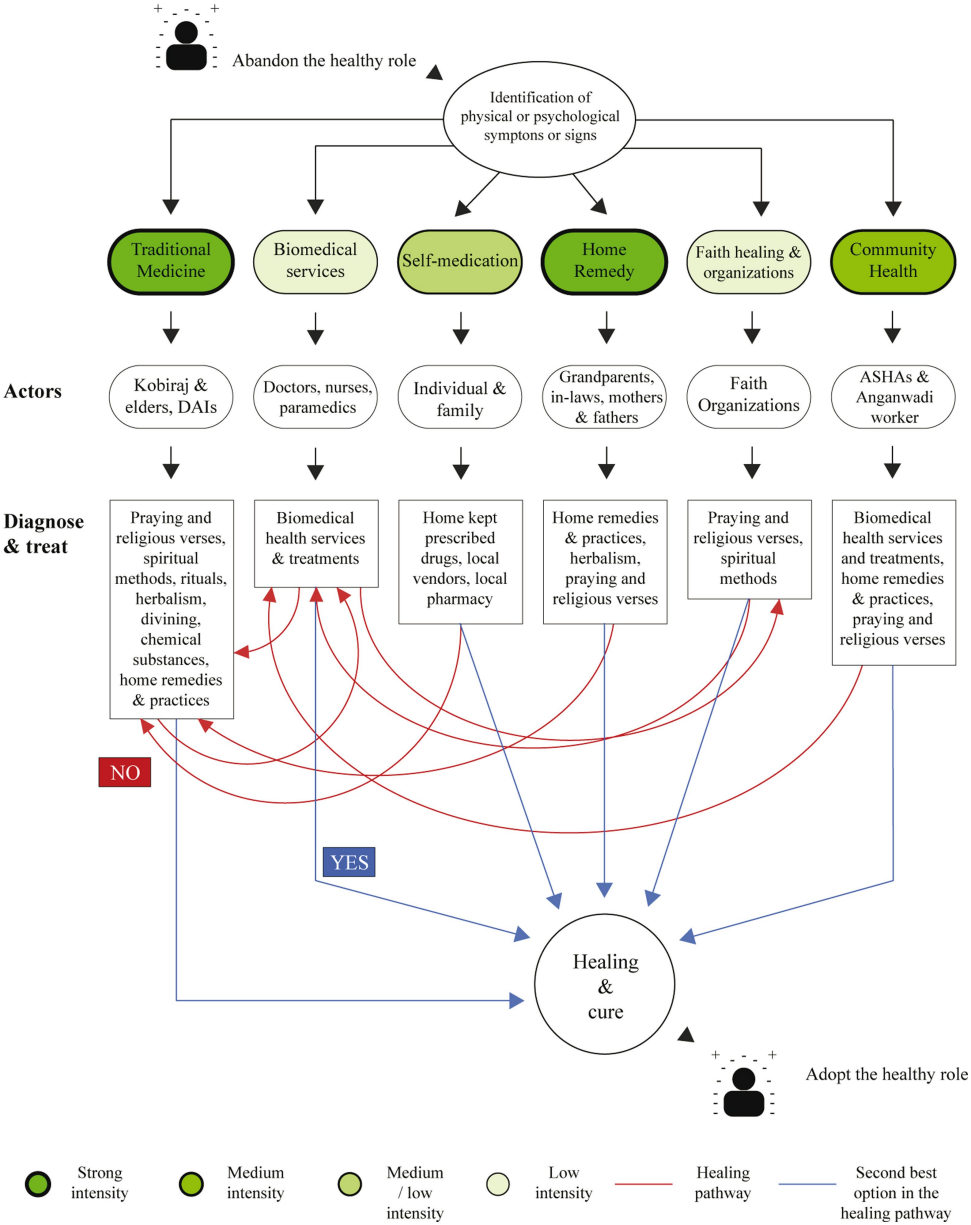
Health-seeking behavior and preferences of the tribe for maternal and newborn health services.

Most participants stated they would access a healthcare facility only when “feeling sick” or when there was a complication or emergency, such as when the woman is “unable to deliver at home”. Participants expressed mixed feelings regarding biomedical services and TM; while some felt health centers were unsafe since appropriate care may not be provided, others thought the TM healers were not trained and thus opted out (Table 2). TM, home remedies, services of the ASHA, and self-medication practices were preferred over accessing biomedical services or even faith-based organizations for their health needs, as illustrated in Figure 3.

**Table 2:** Perceptions toward biomedical services and traditional medicine.

Biomedical services		Interrelated	Traditional medicine	
Positive perceptions	Negative perceptions		Positive perceptions	Negative perceptions
*“The health staff and nurses all take good care (.) they are all from our village. Even the doctor is from our own Liangmai tribe. I can communicate well and share all my problems in my own language, which is very good” – Liangmai woman, 26 years old*	*“We go to hospital ok, some they are wrongly treated, some they die, there are some like that. Go to hospital, wrong medicine is injected.” – Liangmai woman, 27 years old*	*“So, what doctors are not able to treat, I treat it. And what I cannot treat, doctors treat (…) Doctor’s medicines are also good and I believe that it should be used” – Liangmai male traditional healer, 65 years old*	*“How will I know if people trust me, I cannot say it is because I’m like this and that, that is why. But they only believe that by coming here they will be treated and healed” – Liangmai traditional healer, 65 years old*	*“So, for that it will be good if we are seen from the hospitals by the doctors because kobiraj (traditional healer) they have not studied nor are they trained. That’s why, going to the doctors, we feel safe and better” – Liangmai woman, 35 years old*

Gender roles and the extended family played an essential role in influencing HSB.

During pregnancy, there is a strong tendency for women to depend on their male partners, assuming that it is the husband’s responsibility to take care of the woman. Most male participants considered themselves responsible for the delivery as the head of the family (Table 1). However, the health professionals stated that it’s uncommon to see the partners accompanying their wives during the ANC visits. Most husbands admitted to participating in the home delivery by assisting with the birth, cutting the umbilical cord, or burying the placenta. Although the husband’s participation is limited in institutional deliveries mainly due to restrictions imposed in the health facility, they were responsible for facilitating the transportation, communication, and financial arrangements. Extended family (mothers, fathers, grandparents, and in-laws of the mother) also participated during childbirth. Home delivery was a common practice among the elders, and there is a strong belief among the participants that since elders are knowledgeable and experienced, their advice should be followed (Table 1). During home delivery, the family members were summoned to primarily attend to the delivery, take care of the newborn, do the household chores, or occasionally for financial support.

#### The community level

This level moderately influenced the tribe’s HSB, as illustrated in Figure 2. The FGDs identified key community-level actors who influence HSB, such as the ASHAs (CHWs), faith-based organizations (FBO), local health committees, and village councils. The ASHAs were the most influential since most participants referred positively to them. Women considered them as a source of information, advice, emotional and economic support, and primary healthcare providers in their village (Table 1). Some highlighted their importance, as doctors or nurses are usually unavailable in the village. However, most ASHAs and TBAs stated that they were hesitant to help the parturient with home deliveries, especially if the woman is experiencing a “*bad delivery*” (obstetric complications), since they perceive themselves as insufficiently trained to manage complications confidently. They also expressed difficulties in recognizing neonatal conditions.

The IDIs noted that some interviewees were mildly influenced by faith-based organizations, as they are perceived as sources of salvation and healing (Table 1). A few participants even admitted giving birth in prayer centers near their homes. We observed that prior permission from the local church authorities is a prerequisite for conducting any community activity. Almost everyone referred to church leaders with reverence and obedience. Few interviewees regarded Village Health Committees (VHC) and village councils as sources of information but were perceived hierarchically.

#### The organizational and policy level

This level was noted to have the most negligible influence on HSB. At the organizational level, health professionals perceived that their services were underutilized because of the lack of awareness, social mobilization, and proper health facilities; however, they reported no official complaints from the community (Table 1). There was a general lack of awareness of health policies at the policy level, and only a few interviewees referred to the JSSK and JSY programs. The majority of ASHAs reported insufficient and inconsistent payments of the monetary incentives, often paying from their own pockets to cover costs incurred while accompanying the patient. Most interviewees reported attempting to go to the health facility even if the distance was considered a challenge. According to one ASHA, even with the monetary benefits of the JSSK scheme, the mother would need to use additional money to cover travel costs since the villages are far away from the delivery points.

An ASHA also confided in having to confabulate data for the community members to benefit from the financial incentives (Table 1). Most of them were reluctant to promote the schemes since it failed to cover basic expenses.

### Barriers to accessing and utilizing maternal and newborn health services

According to our adapted-three delays model, the most decisive influence was found on the third delay related to the provision of adequate care, which had a negative effect on the first delay associated with the decision to seek healthcare. Consequently, the second delay regarding the arrival at the health facility appeared to be the one with the lowest influence. The results of this model have been summarized and illustrated in Figure 2.

#### Delay in the provision of adequate care

The most relevant factor deterring access to health services is the perceived deficient quality of care in health centers. The limited availability of healthcare professionals, low quality of services/medicines, and irregular facility opening hours were key factors influencing this domain. A typical response noted was that staff are often absent in the facilities. Some interviewees mentioned that the health centers are closed on most days and only functional for a few hours when opened. Respondents also wished the centers were open to conducting deliveries at night (Table 3). The majority perceived that the health centers’ facilities were inadequate as there was a dearth of instruments, supplies, medicines, and equipment. The shortage of essential drugs and supplies in the health centers was corroborated by a doctor, too (Table 3). However, most participants and doctors agreed that vaccines were always available for newborns and mothers.

**Table 3:** Highlights from the focus group discussions and in-depth interviews included in the adapted three-delays model.

Delays	Factors and determinants	Quote
Delay in the provision of adequate care	Services and treatment availability	*“In hospital going and giving birth, our hospital is nothing right? For operations, also no instruments right? For that, operation case comes out, or birth, cannot be normal, like that suffers, for nurses and doctors becomes difficult.” – Liangmai woman, 35 years old*
	Availability of health professionals	*“We are told to go and deliver in the hospitals right? And some they do too when the doctor is present but most of the time they are not present. That’s why since the hospital is here so if the things are complete and everything is done from here then the death also will not be there. Because of the poor facility and improper functioning of it, even the patient’s illness is prolonged right? That’s why I want everything to be there.” – Liangmai woman, 43 years old*
	Quality of the services	*“That is the reason the patients are not coming more. This is also the reason, because in here there are no medical facilities, so we better go outside, sometimes the people used to think like that. So it is not only here, but it is all over Nagaland” – Health professional*
	Opening hours	*“We give birth at home but by nurses… always even though the birth was not from hospital, because the hospital does not open all the time… even if we think of going to the hospital and give birth, it is not open.” – Liangmai woman, 36 years old*
Delay in the decision to seek care	Identification of risks and symptoms	*“When it’s time for delivery and blood comes out, while giving birth, the blood drops. When we get pregnant-heavy, in pregnancy then the baby starts moving. But if the baby does not move then it is to be worried.” – ASHA, 37 years old*
	Health knowledge and self-medication	*“My experience is that some (referring to babies) they have stomach pain and continue crying, so we give Gripes juice ok? If something happens then we take them to hospital also ok? Or if they get jaundice we worry and take to hospital.” – Liangmai husband, 42 years old*
	Decision-making process and preferences	*“But we do not go to the hospital direct, people this side goes to the kobiraj (traditional healer) first. So the kobiraj is first approached.” – Liangmai woman, 31 years old*
		*“So, me if I check and see that it will be hard for me to do, then I send them to the hospital” – Liangmai male traditional healer, 65 years old*
		*“I send them because it is always better to be taken care by someone who knows and have studied right?” – Liangmai female TBA, 58 years old*
	Decision-maker	*“Afterall, the head of the family is me. I should take decisions. So in Zeliangrong or Naga’s context, the head of the family is the man, so decision maker is to be the man only” – Male Liangmai teacher, 38 years old*
Delay in arrival to the facility	Transportation and roads	*“So, roads you just see, from here if you go to (X), it would take around 3 h only, but really it is taking 6 h. So, in 3 h that would save a patient’s life” – Health professional*
	Distance to the health facility	*“In here (X), it is a walkable distance though it is long. But in (X) if we go once. We are ready only for delivery. We cannot come back because it takes a day or so. It is that to travel so far. So, for me and my wife, we do not go to (X) for normal checking” – Liangmai man, 50 years old*
	Health information and sources	*“Other tribes…they have crossed the awareness level now. But, in our level… are still in the awareness. If there is no awareness… everything will be bad” – Liangmai man, Social worker*

#### Delay in the decision to seek care

In this domain, we identified the key factors that influence the decision of a mother to seek healthcare facilities. Most participants preferred home delivery if it was considered an “easy delivery” since it is deemed a familiar and comfortable environment to deliver in (Table 4). Conversely, they perceived complicated obstetric emergencies necessitated an institutional delivery as it is best managed in a health center and may be unsafe at home (Table 4). Specific uncommon or alarming symptoms like retained placenta, non-progress in labor, vaginal bleeding, mal presentations, large abdomen, and swelling triggered the individual or the family to visit healthcare facilities. Newborns were only taken to a health center in case of incessant crying, seizures, jaundice, and any suspected gastrointestinal or respiratory tract infection. A few women and men opted for ANC services offered in health centers, while only some respondents elucidated to seeking postpartum services in health facilities, as homecare was predominantly favored. They perceive visiting a health facility related to sickness, complications, and emergencies and prefer seeking TM first (Table 4). Few women informed that they adopt a vertical position during labor and deliver the baby by kneeling, contrary to what is done in health facilities (Table 4).

**Table 4:** Perceptions toward home delivery and institutional delivery.

Home delivery		Institutional delivery	
Positive perceptions	Negative perceptions	Positive perceptions	Negative perceptions
*“So delivery and delivering at home is the best place. And then the safest place. No hesitation. No new person, no new environment, no new things. So that is the best thing (…) We have many positive things in delivering at home, as I said home is the safest place for the mother, they are really comfortable, they are really acquainted with the things” – Liangmai church worker, 50 years old*	*“Delivering at home, I feel is very dangerous (…) and after torturing your body enough only then give birth, so instead of facing these problems, it is better to go to the hospitals and deliver from there (…) then in hospital, we feel free, no worries. At home it is risky” – Liangmai woman, 38 years old*	*“If we give birth in the hospital it will be good, that’s what I feel because when my children died, that way, I think that might have been while cutting the umbilical cord something went wrong or what. So, it will be better when someone who knows takes care of us, and our children’s life will also be saved” – TBA, 58 years old*	*“Delivery in the hospital, they say: they lay down and deliver but for me I can ‘t deliver by laying down. Those who deliver by laying down their children is good ok? But it is difficult for us. I also feel that laying down and delivering will be difficult, at home I kneel and deliver.” – Liangmai woman, 24 years old*

For most women, TM was the preferred option for primary healthcare due to their availability and since their beliefs and relationships; are assumed to be spiritual or religious. ASHAs, TBAs, and traditional healers informed that they also refer patients to the health centers if they cannot manage an obstetric complication (Table 3). Notably, the TM actors positively perceived the skills of health professionals and considered them “experts” (Table 3). However, health professionals were unfamiliar with TM practices and thus felt disconnected from the TM healers.

Some women indicated that they self-medicate using biomedical medicines on themselves or their babies to avoid visiting a facility. Most interviewees mentioned that they procure the drugs from informal local pharmacies, the local hospital, ASHAs, or local taxi delivery from other villages and stock them at home. Due to the lack of available pediatric preparations, a woman described manually pulverizing tablets and diluting them with hot water to give them to the baby. Women agreed that the husband is the decision-maker and his permission regarding health decisions is essential; however, this was not stated as a rule (Table 3).

#### Delay in arrival at the facility

Three significant factors were identified under this domain: transportation and roads, distance to the health facility, and health information and sources (Table 3). The dilapidated condition of the roads appeared to be the most influential factor, as the majority of respondents, specifically men, mentioned challenges in arranging a vehicle to transport patients due to bad roads. Some preferred to travel by foot, and some had either experienced or heard about women delivering on the way. The price quoted by private transportation agencies was often higher than the average costs, especially after dark, increasing the OOP expenditure incurred due to the lack of alternatives like ambulance services or public transportation. An ASHA disclosed that the incentive received to cover transportation costs in the JSSK scheme did not suffice in these circumstances.

The female participants informed that the ASHA was the primary source for information on the services provided in the health centers and health in general. Few respondents indicated receiving information from nurses and doctors, while others received it from the TBAs. A general sense of lack of awareness in their tribe compared to other tribes was conveyed (Table 3).

We visited three health centers located in and around our study villages to corroborate the findings from the IDIs and FGDs. One was open, had an ambulance, and implemented a functional newborn care corner in the labor room. Multiple educational posters and signs of maternal and newborn health were available, informing about immunizations, ANC, PNC, clean delivery practices, governmental incentives like JSY and JSSK, family planning, breastfeeding, malaria and HIV awareness, newborn resuscitation, and the ASHA program. However, almost all the information was presented in English, not in Liangmai. The other facility we visited was sub-optimally functional, with non-availability of running water, improper biohazardous waste disposal, fluctuant electricity, and a lack of modern medical equipment. Some medicines were expired, and others were not properly stored. Supplies seemed to be lacking in the delivery room too. No patient was in the out-patient or in-patient ward during the observation. One visited facility was closed and not functional apparently for a long time.

## Discussion

In this concurrent mixed-methods study, we studied the HSB of the mothers and women from the Liangmai tribe and the factors that influence them. To our knowledge, this is the first study to be conducted in this region concerning this topic. The maternal HSB exhibited by this community was influenced by several social, economic, cultural, geographic, and political factors that were closely interlinked.

We identified that the interpersonal and intrapersonal factors significantly influenced the HSB of this tribe. As their perception of risk was low, the tribe has an emergency-driven health-seeking behavior only with evident physical signs of danger. Thus, there is a low preventive-driven health-seeking behavior for ANC and PNC (21). This explains why only 4% of women reported four routine antenatal visits and 41% delivered at home. Functional health centers were better utilized for deliveries by locals living in that village than the villages with no or non-functional health centers. Another dominant reason for low healthcare facility utilization was that community members physically accessed biomedical services only when they perceived severe sickness, complications, or an emergency. It was sought secondary to failure of primary treatment with TM, home remedy, or self-medication. Similar findings were reported in a study of the socio-cultural and service delivery dimensions of maternal mortality in central India (22).

Since pregnancy for this tribe is a normal process and primarily not associated with a disease or a health problem, the decision to visit a health facility is often limited in childbirth, which is additionally determined by obstetric emergencies or in moments of fear. A similar emergency-driven HSB for newborn conditions was observed, except for seeking preventive health associated with receiving immunizations, perhaps because vaccines were most often available in the health centers. This protective behavior can be leveraged as an entry point for mothers and babies to access other health services. Since access to a health center is challenging in this arduous terrain and preventive health is not a priority to this community. Routine preventive antenatal, postnatal, and immunization services could alternatively be delivered monthly by a mobile medical unit linked with the health center allocated for that area in collaboration with the ASHAs as implemented successfully in other Indian states like Tamil Nadu (23).

Previous cases of death of the mother or newborn during childbirth influenced the HSB of the individuals, especially since infant mortality and spontaneous abortions were commonly reported and complemented by the survey results. This contradicts the governmental statistics, where only four stillbirths were reported in the district from 2018 to 19 (17). Any health event occurring in a healthcare facility is more likely to be captured formally by the health information system, but since access to these facilities is limited in remote tribal areas, deaths, and events occurring in these villages are likely to be missed. A national-level survey in India revealed that out of 10,109 scheduled tribe women, 44% reported difficulties accessing healthcare services because of the distance to the health facility and 42% due to inadequate transportation options (24). The results from the multivariable logistic regression analysis also supported this by suggesting that mothers residing in the villages without a health center or one that was not operational were more likely to deliver at home, suggesting that challenges in access may influence the decision to deliver at home.

Traditions and practices handed down from generation to generation influenced the current behavior of the tribe, which still firmly believes in faith healing. This belief is reinforced since TM healers are always locally available, accessible, and conforming to their religious beliefs. Therefore, culture determines the perceptions of the individual and the community regarding their definition of health and well-being (25). This also influences or shapes their sense of coherence, initiating or stopping the individual on the health-ease-disease continuum. In a medical pluralistic system, the Liangmais are struggling between traditional practices and the biomedical health system representing a mixed, interrelated, interdependent, and in a way, competing structure.

From a non-cognitive perspective, the individual’s socioeconomic status influences their decision to seek healthcare services, as highlighted by other studies conducted in Bangladesh and Africa (26–29). The direct and indirect costs incurred to access public services are strongly associated with considerable personal expenditure since the services are not perceived as free. Therefore, they chose to stick to their traditions and prioritize other financially viable options, such as TM or home remedies, as biomedical health expenditures pose a high economic burden on their families. Another study conducted in India also reported financial barriers associated with the lack of access to ANC and safe delivery with lower socioeconomic characteristics (4). The Government of Nagaland has also acknowledged this high OOP expenditure experienced by patients in the state in the State Human Development Report (7). Nevertheless, our observations indicate that in emergency situations, when alternative local healthcare options have been exhausted, financial resources do not play a determining role in visiting healthcare facilities.

When analyzing the adapted three-delays model, we noticed that a delay in one level significantly affects the other subsequent levels. The delay in providing adequate care strongly enhances the uncertainty in the decision to seek care with moderate influence from the delay in arrival to the facility, thus impeding people of the tribe from effectively and promptly seeking biomedical healthcare. Nevertheless, the community finds alternatives to the presented barriers by accessing TM actors, self-medicating using biomedical drugs, or home remedies. As observed in various medical pluralistic studies conducted in South America, a combination of the three alternate modalities has also been demonstrated by the Liangmais (30–32). Since access to essential biomedical medication in the village and health centers is difficult, women tend to store medicines at home and adopt various dosage mechanisms for their babies. Pregnant women and their babies risk experiencing untoward side effects or overdosage due to unsupervised self-medication, as described in a study conducted among pregnant women from Iran (33).

The delay in providing adequate care was also attributed to all previous individual or community experiences, rumors, and beliefs related mainly to the low quality of the services available in the health facilities. Our findings corroborated with *Singh* et al.*’s* findings that the lack of resources and services in health centers affects institutional delivery rates in Northeast India (34). Significant concerns regarding the availability of healthcare professionals, opening hours, and lack of medicines and health services deterred them from even attempting to seek maternal or newborn biomedical healthcare services. This was complemented by a study conducted in rural Nigeria that reported that low utilization of government facilities was due to staff irregularities, low quality of services, and the high costs incurred to access them (35). Even health professionals agreed that the facilities were not offering adequate services, which demotivates them from promoting the services, obliging them to adopt risky treatment options and plan strategies for emergency referrals. Given the community’s perception that the quality of services provided at the health centers is defective, the conventional approach to increase the overall knowledge to improve HSB in these communities may prove futile unless the quality and quantity of services in the health centers improve. The state has launched a World Bank-aided project to strengthen a few health centers to mitigate this issue (36). However, this impact is yet to be ascertained in the few communities they serve.

The generalized feeling of shyness among women was an essential attitudinal determinant that discouraged them from consulting male doctors and visiting the health facility since they associate biomedical practices with examining reproductive and sexual organs. Similar findings where feelings of embarrassment influenced the HSB of mothers from Amazonian communities in Peru were reported by Westgard et al. (37). The gender of the healthcare provider (female TBAs, ASHAs, or nurses) is preferred during childbirth. However, this cannot be assumed as the only factor, as male relatives attended deliveries, also denoting a complementary behavior that prefers trust or familiar relationships over gender preferences.

There is no kinship or formal communication between the doctors and the traditional actors, which explains the misunderstandings or incompatibilities between the two systems. It can be assumed that reluctance toward TM originates from the health professional’s perspective. Nonetheless, TM actors see biomedicine, to some extent, as complementary. Some of these elements are also stated in a study of medical pluralism and its implications on health policy in Northeast India (38). Similarly, a rural Ghana study showed that health personnel did not consider traditional birth attendants’ contribution (39). There is also a weak relationship between the community and the doctors, as doctors are not perceived to be present in the facilities when needed. However, a better relationship exists between the community and the nurses, especially for delivery. The most substantial relationship was noted between the ASHAs and the women, especially in the village with a non-functional health center as corroborated by another study from India (40). ASHAs are perceived to have gained credibility in the community, leading to social recognition and increased legitimacy as service providers. Consequently, this kinship can be a potential mechanism to reduce maternal and newborn mortality and morbidity, considering community-oriented primary healthcare as an essential strategy to reduce inequalities by adequately training them and proportionately officially recognizing their efforts and health work, especially in rural, remote indigenous areas such as this. In addition to the ASHAs, traditional healers and TBAs are also primary contact points for mothers seeking treatment. They can also be sensitized and engaged in disseminating information regarding preventive health and be trained to recognize red flags to initiate early referrals.

Another approach to address the low uptake of health services is to strengthen a process named “Communitization,” which formulates a Health Centre Managing Committee (HCMC) that consists of members from the village council, FBOs, and staff from the health center located in the village to closely monitor the functioning of the health center along with the Government. Even before being endorsed nationwide by the National Health Mission, this system originated from a policy implemented by the Government of Nagaland in 2002, titled *“The Nagaland Communization of Public Institutions and Services Act”* (40). The health component of the scheme purports to ensure that the community is sufficiently and meaningfully represented and empowered to participate in the health center’s planning, implementation, and utilization of services. The HCMC can be further empowered to monitor the performance of government schemes that are intended to influence healthcare delivery uptake positively. The impact and challenges faced by HCMCs in Nagaland have been comprehensively documented by Tushi and Kaur (41).

Our study highlights that if the perceived basic needs of the community are unmet, all other efforts become irrelevant, and services will not be utilized satisfactorily by the community. In a setting such as this, where the community functions around socio-cultural norms, it may be pragmatic for policymakers to adopt a “bottom-up approach” and tailor programs by placing the community’s needs before national objectives. Policies must focus on primarily empowering local village councils, FBOs, and VHCs to monitor their health centers effectively. Inter-sectoral policies from the educational, economic, employment, infrastructure, housing, sanitation, and essential services must also be considered to address the social determinants contributing to this issue. It is relevant to design and implement salutogenic enabling environments that allow individuals to make informed decisions about their health. The objective is to attain health and well-being by guaranteeing substantive freedom and respect for the self-determination of indigenous people.

## Conclusion

The HSB of this community is influenced by multiple social, economic, cultural, geographic, and political factors that are closely related. Health facilities are sought only in emergencies, secondary to locally available traditional medicine, determined primarily by the tribe’s perception of the quality of services provided and challenges in geographical access.

Economic reasons, low awareness, unrealistic reimbursements, and inadequate implementation of national schemes were pivotal in deterring access to health facilities. Engaging, recognizing, integrating, and supporting local actors like ASHAs, TBAs, TM healers, and FBOs may be a more realistic approach to influence individual or familial decisions to seek health facilities for maternal and child health. Community agents trained to consider the needs of vulnerable population groups represent a key catalyst in the community system. In addition, community perspectives can provide crucial insight into why health interventions may struggle or succeed in various locations. Moreover, community-led monitoring can provide a vital tool for accountability. This framework must also be supported with adequate financing for these community systems.

At the policy level, the two national programs to financially incentivize institutional deliveries and decrease OOP expenses failed to impact the local health center utilization rates due to inadequate program implementation at the grassroots as it did not consider this region’s cultural and geographical differences. The amount intended to incentivize mothers and ASHAs failed to cover the basic costs they incurred to reach a delivery point. The “one size fits all” approach to planning these policies implemented countrywide has to be tailored to the local needs of the region and must factor in the exorbitant direct and indirect costs involved with travel on rugged terrains.

Strengthening and empowering existing Health Centre Managing Committees to monitor their health centers may improve service quality. The crucial role and impact of communities stem from their strengths, which include but are not limited to legitimacy among individuals, a strong understanding of the contexts, trust, social solidarity, social motivation, and adaptation abilities. Community systems offer critical support in the areas where the conventional health system fails. The extent to which the terrain influences the delays in healthcare access should be comprehensively studied.

### Study strengths and limitations

This study is strengthened by its mixed-method approach, reinforcing its implementation with community actors, empowering people as agents who can investigate their own situation, and providing an alternative to traditional research, especially in remote and indigenous areas. The quantitative evidence from a community-based survey was supported by the findings that evolved from the qualitative components of this marginalized population. This study was conducted in one of the region’s most remote areas, being indicative of logistical problems and organizational/financial difficulties.

Due to time constraints, 22 of the 26 audio recordings were directly transcribed and translated to English, with additional revisions of the translations in collaboration with an external Liangmai native speaker.

## Data availability statement

The raw data supporting the conclusions of this article will be made available by the authors, without undue reservation.

## Ethics statement

The studies involving human participants were reviewed and approved by the Ethics Committee of the Medical Faculty, the Ruprecht Karls University of Heidelberg, Germany (#S-138/2019, 13.03.2019), and the Institutional Review Board (Research and Ethics Committee) of the Christian Institute of Health Sciences and Research, Nagaland, India. Written informed consent was obtained from the individual(s) for the publication of any potentially identifiable images or data included in this article.

## Author contributions

AD, ÁLC, RK, and RR developed the idea and designed the study. ÁLC and RR elaborated the methodology and data collection tools with the support of AD. ÁLC, PN, and RK collected the data. PN translated and transcribed the data. ÁLC analyzed the qualitative data with the backing of AD. RR analysed the quantitative data. ÁLC, RR, PN, RK, and AD contributed to data interpretation. ÁLC drafted the manuscript. ÁLC, AD, and RR revised the final version of this manuscript. All authors contributed to the article and approved the submitted version.

## Funding

A research grant from the German Academic Exchange Service (DAAD) covered logistical costs for the fieldwork. All the other costs were covered by ÁLC. The Science and Engineering Research Board, Department of Science and Technology (Sanction Order: ECR/2016/002059), funded the community-based survey (Project on the use of Geographic Information Systems in Health Systems Research in Rural Areas of Nagaland). For the publication fee we acknowledge financial support by Deutsche Forschungsgemeinschaft within the funding programme “Open Access Publikationskosten” as well as by Heidelberg University.

## Conflict of interest

The authors declare that the research was conducted in the absence of any commercial or financial relationships that could be construed as a potential conflict of interest.

## Publisher’s note

All claims expressed in this article are solely those of the authors and do not necessarily represent those of their affiliated organizations, or those of the publisher, the editors and the reviewers. Any product that may be evaluated in this article, or claim that may be made by its manufacturer, is not guaranteed or endorsed by the publisher.

## Abbreviations

ASHA: Accredited social health activist
ANC: Antenatal care
CHW: Community health worker
TBA: Traditional birth attendant
FGD: Focus group discussion
HSB: Health seeking behavior
IDI: In-depth interviews
JSSK: Janani Shishu Suraksha Karyakaram
JSY: Janani Suraksha Yojana
OOP: Out-of-pocket expenditure
PNC: Postnatal care
TH: Traditional healers
TM: Traditional medicine
SEM: Socio-ecological model
