# Supplementary material for: Perceptions, health seeking behavior and utilization of maternal and newborn health services among an indigenous tribal community in Northeast India—a community-based mixed methods study
Source: Front Public Health. 2023 Jul 6;11:1139334. doi: 10.3389/fpubh.2023.1139334 (PMC10358725; doi:10.3389/fpubh.2023.1139334)
Supplement: Supplementary file 1 [file Table_1.docx]

Supplementary Material

**Supplementary Table 1: Background characteristics of the indigenous tribal mothers who participated in the community-based survey.**

|  | **Total** | |  | **Village 1^#^**  **(n=24)** | |  | **Village 2^#^**  **(n=62)** | |  | **Village 3^#^**  **(n=23)** | | |  | |  | | **Chi-square ______** | |
| --- | --- | --- | --- | --- | --- | --- | --- | --- | --- | --- | --- | --- | --- | --- | --- | --- | --- | --- |
|  | ***n*** | ***%*** |  | ***n*** | ***%*** |  | ***n*** | ***%*** |  | ***n*** | | ***%*** |  | |  | | ***P-*value** | |
| **Age (n=109)** | | | | | | | | | | | | | | | | | | |
| 15-19 years | 3 | 2.8 |  | 1 | 4.2 |  | 2 | 3.2 |  | 0 | 0.0 | |  |  | | 0.38* | |  |
| 20-29 years | 80 | 73.4 |  | 21 | 87.5 |  | 41 | 66.1 |  | 18 | 78.3 | |  |  | |  | |  |
| 30-39 years | 23 | 21.1 |  | 2 | 8.3 |  | 16 | 25.8 |  | 5 | 21.7 | |  |  | |  | |  |
| 40-49 years | 3 | 2.8 |  | 0 | 0.0 |  | 3 | 4.8 |  | 0 | 0.0 | |  |  | |  | |  |
| **Marital status (n=109)** | | | | | | | | | | | | | | | | | | |
| Married | 108 | 99.1 |  | 23 | 95.8 |  | 62 | 100 |  | 23 | | 100 |  | |  | | 0.43* | |
| Unmarried | 1 | 0.9 |  | 1 | 4.2 |  | 0 | 0.0 |  | 0 | | 0.0 |  | |  | |  | |
| **Education (n=109)** | | | | | | | | | | | | | | | | | | |
| Nil | 1 | 0.9 |  | 0 | 0.0 |  | 1 | 1.6 |  | 0 | | 0.0 |  | |  | | 0.12* | |
| 1-5 grades (Primary school) | 23 | 21.1 |  | 7 | 29.2 |  | 9 | 14.5 |  | 7 | | 30.4 |  | |  | |  | |
| 5-8 grades (Middle school) | 51 | 46.8 |  | 14 | 58.3 |  | 25 | 40.3 |  | 12 | | 52.2 |  | |  | |  | |
| 9-10 grades (High school) | 20 | 18.3 |  | 2 | 8.3 |  | 14 | 22.6 |  | 4 | | 17.4 |  | |  | |  | |
| 11-12 grades (Higher secondary school) | 8 | 7.3 |  | 0 | 0.0 |  | 8 | 12.9 |  | 0 | | 0.0 |  | |  | |  | |
| Graduate | 6 | 5.5 |  | 1 | 4.2 |  | 5 | 8.1 |  | 0 | | 0.0 |  | |  | |  | |
| **Occupation (n=109)** | | | | | | | | | | | | | | | | | | |
| Community health worker | 4 | 3.7 |  | 0 | 0.0 |  | 4 | 6.5 |  | 0 | | 0.0 |  | |  | | **<0.01*** | |
| Farmer | 45 | 41.3 |  | 19 | 79.2 |  | 12 | 19.4 |  | 14 | | 60.9 |  | |  | |  | |
| Housewife | 50 | 45.9 |  | 3 | 12.5 |  | 38 | 61.3 |  | 9 | | 39.1 |  | |  | |  | |
| Teacher | 2 | 1.8 |  | 0 | 0.0 |  | 2 | 3.2 |  | 0 | | 0.0 |  | |  | |  | |
| Shop keeper | 4 | 3.7 |  | 0 | 0.0 |  | 4 | 6.5 |  | 0 | | 0.0 |  | |  | |  | |
| Others | 4 | 3.7 |  | 2 | 8.4 |  | 2 | 3.2 |  | 0 | | 0.0 |  | |  | |  | |
| **ANC check at a health centre (n=109)** | | | | | | | | | | | | | | | | | | |
| Nil | 13 | 11.9 |  | 7 | 29.2 |  | 3 | 4.8 |  | 3 | | 13.0 |  | |  | | **<0.01*** | |
| 1 | 16 | 14.7 |  | 11 | 45.8 |  | 3 | 4.8 |  | 2 | | 8.7 |  | |  | |  | |
| 2 | 52 | 47.7 |  | 5 | 20.8 |  | 42 | 67.7 |  | 5 | | 21.7 |  | |  | |  | |
| 3 | 24 | 22.0 |  | 1 | 4.2 |  | 13 | 21.0 |  | 10 | | 43.5 |  | |  | |  | |
| ≥4 | 4 | 3.7 |  | 0 | 0 |  | 1 | 1.6 |  | 3 | | 13.0 |  | |  | |  | |
| **Gravida (n=109)** | | | | | | | | | | | | | | | | | | |
| 1 | 24 | 22.0 |  | 4 | 16.7 |  | 18 | 29.0 |  | 2 | | 8.7 |  | |  | | 0.08* | |
| 2 | 24 | 22.0 |  | 4 | 16.7 |  | 12 | 19.4 |  | 8 | | 34.8 |  | |  | |  | |
| 3 | 22 | 20.2 |  | 2 | 8.3 |  | 14 | 22.6 |  | 6 | | 26.1 |  | |  | |  | |
| 4 | 15 | 13.8 |  | 7 | 29.2 |  | 5 | 8.1 |  | 3 | | 13.0 |  | |  | |  | |
| ≥5 | 24 | 22.0 |  | 7 | 29.2 |  | 13 | 21.0 |  | 4 | | 17.4 |  | |  | |  | |
| **Living children (n=109)** | | | | | | | | | | | | | | | | | | |
| No living children | 24 | 22.0 |  | 4 | 16.7 |  | 18 | 29.0 |  | 2 | | 8.7 |  | |  | | 0.40* | |
| 1 | 5 | 4.6 |  | 1 | 4.2 |  | 2 | 3.2 |  | 2 | | 8.7 |  | |  | |  | |
| 2 | 26 | 23.9 |  | 3 | 12.5 |  | 14 | 22.6 |  | 9 | | 39.1 |  | |  | |  | |
| 3 | 21 | 19.3 |  | 5 | 20.8 |  | 12 | 19.4 |  | 4 | | 17.4 |  | |  | |  | |
| 4 | 16 | 14.7 |  | 5 | 20.8 |  | 7 | 11.3 |  | 4 | | 17.4 |  | |  | |  | |
| ≥5 | 17 | 15.6 |  | 6 | 25.0 |  | 9 | 14.5 |  | 2 | | 8.7 |  | |  | |  | |
| **Previous abortions reported by multigravida mothers (n=85)** | | | | | | | | | | | | | | | | | | |
| No reported abortions | 76 | 89.4 |  | 0 | 0 |  | 55 | 88.7 |  | 21 | | 91.3 |  | |  | | 0.61* | |
| 1 | 6 | 7.1 |  | 0 | 0 |  | 5 | 8.1 |  | 1 | | 4.3 |  | |  | |  | |
| 2 | 3 | 3.5 |  | 0 | 0 |  | 2 | 3.2 |  | 1 | | 4.3 |  | |  | |  | |
| **Previous still births/infant deaths (n=109)** | | | | | | | | | | | | | | | | | | |
| No reported deaths | 93 | 85.3 |  | 18 | 75.0 |  | 52 | 83.9 |  | 23 | | 100 |  | |  | | 0.27* | |
| 1 | 12 | 11.3 |  | 6 | 25.0 |  | 6 | 9.7 |  | 0 | | 0 |  | |  | |  | |
| 2 | 4 | 3.7 |  | 0 | 0 |  | 4 | 6.5 |  | 0 | | 0 |  | |  | |  | |
| **Place of delivery (n=109)** | | | | | | | | | | | | | | | | | | |
| Public district hospitals | 15 | 13.8 |  | 1 | 4.2 |  | 12 | 19.4 |  | 2 | | 8.7 |  | |  | | **<0.01*** | |
| Private hospitals | 5 | 4.6 |  | 0 | 0 |  | 5 | 8.1 |  | 0 | | 0 |  | |  | |  | |
| Public primary health centres | 45 | 41.3 |  | 10 | 41.7 |  | 31 | 50.0 |  | 4 | | 17.4 |  | |  | |  | |
| Home | 44 | 40.4 |  | 13 | 54.2 |  | 14 | 22.6 |  | 17 | | 73.9 |  | |  | |  | |
| **Person responsible for the delivery (n=109)** | | | | | | | | | | | | | | | | | | |
| ASHA | 11 | 10.1 |  | 2 | 8.3 |  | 1 | 1.6 |  | 8 | | 34.8 |  | |  | | **<0.01*** | |
| Doctor | 2 | 1.8 |  | 0 | 0 |  | 2 | 3.2 |  | 0 | | 0 |  | |  | |  | |
| Nurse | 70 | 64.2 |  | 10 | 41.7 |  | 52 | 83.9 |  | 8 | | 34.8 |  | |  | |  | |
| Relative | 25 | 22.9 |  | 12 | 50.0 |  | 7 | 11.3 |  | 6 | | 26.1 |  | |  | |  | |
| TBA | 1 | 0.9 |  | 0 | 0 |  | 0 | 0 |  | 1 | | 4.3 |  | |  | |  | |
| **Fully immunized children >9 months of age** (n=67)** | | | | | | | | | | | | | | | | | | |
| Yes | 50 | 74.6 |  | 9 | 69.2 |  | 25 | 65.8 |  | 16 | | 100 |  | |  | | **0.01*** | |
| No | 17 | 25.4 |  | 4 | 30.8 |  | 13 | 34.2 |  | 0 | | 0 |  | |  | |  | |

* Fisher’s exact test

**Fully immunized: all children above > 9 months of age who received BCG, 3 doses each of Oral Polio, DPT, and Measles vaccine.

#Three villages from the Tening block in Peren district were selected based on the presence and functionality of a proximal primary health center. Village 1: has no health facility, Village 2: has a functional health facility and Village 3 with a non-operational health facility.

**Abbreviations:** ASHA- Accredited social health activist, TBA- Traditional birth attendant, ANC- Antenatal care

**Supplementary Table 2: Demographic attributes of the participants of the FGDs and IDIs.**

|  | **Total** | |  | **Females** | |  | **Males** | | |
| --- | --- | --- | --- | --- | --- | --- | --- | --- | --- |
|  | **(N=57)** | |  | **(N=35)** | |  | **(N=22)** | | |
|  | ***n*** | ***%*** |  | ***n*** | ***%*** |  | ***n*** | ***%*** | |
| **Age (n=53)** | | | | | | | | |  |
| 21-25 years | 10 | 17.5 |  | 9 | 25.7 |  | 1 | 4.5 | |
| 26-30 years | 10 | 17.5 |  | 7 | 20.0 |  | 3 | 13.6 | |
| 31-35 years | 12 | 21.1 |  | 9 | 25.7 |  | 3 | 13.6 | |
| 36-40 years | 7 | 12.3 |  | 4 | 11.4 |  | 3 | 13.6 | |
| 41-45 years | 5 | 8.8 |  | 3 | 8.6 |  | 2 | 9.1 | |
| 46-51 years | 4 | 7.0 |  | 1 | 2.9 |  | 3 | 13.6 | |
| ≥51 years | 5 | 8.8 |  | 2 | 5.7 |  | 3 | 13.6 | |
| Missing | 4 | 7.0 |  | 0 | 0 |  | 4 | 18.2 | |
| **Marital status (n=56)** | | | | | | | | |  |
| Married | 44 | 77.2 |  | 28 | 80.0 |  | 16 | 72.7 | |
| Single | 10 | 17.5 |  | 5 | 14.3 |  | 5 | 22.7 | |
| Separated | 1 | 1.8 |  | 1 | 2.9 |  | 0 | 0.0 | |
| Widowed | 1 | 1.8 |  | 1 | 2.9 |  | 0 | 0.0 | |
| Missing | 1 | 1.8 |  | 0 | 0.0 |  | 1 | 4.5 | |
| **Education (n=53)** | | | | | | | | |  |
| 1-5 (Primary school) | 25 | 43.9 |  | 21 | 60.0 |  | 4 | 18.2 | |
| 5-8 (Middle school) | 7 | 12.3 |  | 4 | 11.4 |  | 3 | 13.6 | |
| 9-10 (High school) | 10 | 17.5 |  | 2 | 5.7 |  | 8 | 36.4 | |
| 11-12 (Higher secondary) | 2 | 3.5 |  | 1 | 2.9 |  | 1 | 4.5 | |
| Graduate | 9 | 15.8 |  | 5 | 14.3 |  | 4 | 18.2 | |
| Missing | 4 | 7.0 |  | 2 | 5.7 |  | 2 | 9.2 | |
| **Occupation (n=52)** | | | | | | | | |  |
| Farmer | 21 | 36.8 |  | 12 | 34.3 |  | 9 | 40.9 | |
| Housewife | 7 | 12.3 |  | 7 | 20.0 |  | 0 | 0.0 | |
| Teacher | 2 | 3.5 |  | 0 | 0.0 |  | 2 | 9.1 | |
| Church worker | 2 | 3.5 |  | 0 | 0.0 |  | 2 | 9.1 | |
| Artisan | 5 | 8.8 |  | 5 | 14.3 |  | 0 | 0.0 | |
| Others | 15 | 26.3 |  | 11 | 31.4 |  | 4 | 18.2 | |
| Missing | 5 | 8.8 |  | 0 | 0.0 |  | 5 | 22.7 | |

**Supplementary Table 3:** **Multivariable analysis of factors associated with home delivery among the indigenous tribal mothers in the community-based survey**

|  | **Total** |  | **Home Delivery** | | |  | | **Institutional Delivery** | | |  | | **Univariable** | | | |  | | **Multivariable (full model)** | | | | |  | | | **Multivariable (backward selection)** | | | |
| --- | --- | --- | --- | --- | --- | --- | --- | --- | --- | --- | --- | --- | --- | --- | --- | --- | --- | --- | --- | --- | --- | --- | --- | --- | --- | --- | --- | --- | --- | --- |
|  | **(n=109) (%=100)** |  | **(n=44)** | | |  | | **(N=65)** | | |  | |  |  |  |  |  | |  |  |  |  |  |  | | |  |  |  |  |
|  | ***n*** |  | ***n*** | ***%*** |  | | ***n*** | | ***%*** |  | | ***P-* value** | | **uOR** | **95% CI** |  | | **aOR** | | | **95% CI** | |  | | | **aOR** | | | **95% CI** | |
| **Age^#^** | | | | | | | | | | | | | | | | | | | | | | | | | | | | | | |
| ≥25 years | 71 |  | 28 | 39.4 |  | | 43 | | 60.6 |  | | 0.80 * | | 0.9 | 0.4-2.0 |  | | | | 1.3 | | 0.4-4.0 | | |  | | | – | | – |
| <25 years | 38 |  | 16 | 42.1 |  | | 22 | | 57.9 |  | |  | | ref |  |  | | | | ref | |  | | |  | | |  | |  |
| **Education^#^** | | | | | | | | | | | | | | | | | | | | | | | | | | | | | | |
| Middle school and below | 75 |  | 36 | 48.0 |  | | 39 | | 52.0 |  | | **0.02*** | | **3.0** | **1.2-7.5** |  | | 0.6 | | | 0.2-1.8 | |  | | | – | | | – | |
| High school and above | 34 |  | 8 | 23.5 |  | | 26 | | 76.5 |  | |  | | ref |  |  | | ref | | |  | |  | | |  | | |  | |
| **Occupation** | | | | | | | | | | | | | | | | | | | | | | | | | | | | | | |
| Housewives | 50 |  | 20 | 40.0 |  | | 30 | | 60.0 |  | | 0.90* | | 0.1 | 0.5-2.1 |  | | 2.4 | | | 0.8-7.5 | |  | | | – | | | – | |
| Others | 59 |  | 24 | 40.7 |  | | 35 | | 59.3 |  | |  | | ref |  |  | | ref | | |  | |  | | |  | | |  | |
| **ANC check at a health centre^#^** | | | | | | | | | | | | | | | | | | | | | | | | | | | | | | |
| ≤1 | 29 |  | 15 | 51.7 |  | | 14 | | 48.3 |  | | 0.15* | | 1.9 | 0.8-4.5 |  | | 0.9 | | | 0.3-2.9 | |  | | | – | | | – | |
| 2–4 | 80 |  | 29 | 36.2 |  | | 51 | | 63.8 |  | |  | | ref |  |  | | ref | | |  | |  | | |  | | |  | |
| **Gravida** | | | | | | | | | | | | | | | | | | | | | | | | | | | | | | |
| Multi | 85 |  | 39 | 45.9 |  | | 46 | | 54.1 |  | | **0.03**** | | **3.2** | **1.1-9.4** |  | | 2.0 | | | 0.5-8.3 | |  | | | – | | | – | |
| Primi | 24 |  | 5 | 20.8 |  | | 19 | | 79.2 |  | |  | |  |  |  | |  | | |  | |  | | |  | | |  | |
| **Living children^#^** | | | | | | | | | | | | | | | | | | | | | | | | | | | | | | |
| ≥3 | 54 |  | 25 | 46.3 |  | | 29 | | 53.7 |  | | 0.20* | | 1.6 | 0.8-3.5 |  | | 0.7 | | | 0.2-2.3 | |  | | | – | | | – | |
| <3 | 55 |  | 19 | 34.5 |  | | 36 | | 65.5 |  | |  | | ref |  |  | | ref | | |  | |  | | |  | | |  | |
| **Previous abortion** | | | | | | | | | | | | | | | | | | | | | | | | | | | | | | |
| No abortion | 100 |  | 43 | 43.0 |  | | 57 | | 57.0 |  | | 0.08** | | 6.0 | 0.7-50.1 |  | | 0.1 | | | 0.01-1.6 | |  | | | – | | | – | |
| ≥1 | 9 |  | 1 | 11.1 |  | | 8 | | 88.9 |  | |  | | ref |  |  | | ref | | |  | |  | | |  | | |  | |
| **Previous still births/infant deaths** | | | | | | | | | | | | | | | | | | | | | | | | | | | | | | |
| No deaths | 93 |  | 38 | 40.9 |  | | 55 | | 59.1 |  | | 0.80* | | 1.2 | 0.4-3.4 |  | | 1.1 | | | 0.3-4.0 | |  | | | – | | | – | |
| 1–2 | 16 |  | 6 | 37.5 |  | | 10 | | 62.5 |  | |  | | ref |  |  | | ref | | |  | |  | | |  | | |  | |
| **Village^$^** | | | | | | | | | | | | | | | | | | | | | | | | | | | | | | |
|  |  |  |  |  |  | |  | |  |  | |  | |  |  |  | |  | | |  | |  | | |  | | |  | |
| Village 1 | 24 |  | 13 | 22.6 |  | | 11 | | 77.4 |  | | **<0.05*** | | **4.1** | **1.5-11.0** |  | | **5.0** | | | **1.1-23.1** | |  | | | **4.1** | | | **1.5-11.0** | |
| Village 3 | 23 |  | 17 | 73.9 |  | | 6 | | 26.1 |  | | **<0.01*** | | **9.7** | **3.2-29.3** |  | | **12.4** | | | **3.1-49.0** | |  | | | **9.7** | | | **3.2-29.3** | |
| Village 2 | 62 |  | 14 | 54.2 |  | | 48 | | 45.8 |  | |  | | **ref** |  |  | | **ref** | | |  | |  | | | **ref** | | |  |  |

*Chi² test

**Fisher’s exact test

# Classified using median cutoffs

$ Three villages from the Tening block in Peren district were selected based on the presence and functionality of a proximal primary health centre. Village 1: has no health facility, Village 2: has a functional health facility and Village 3 with a non-functional health facility.

**Abbreviations:** uOR- unadjusted odds ratio, aOR- adjusted odds ratio, CI- confidence interval, ANC- Antenatal care, ref- reference
